# Supplementary figures and images for: Activation of targetable inflammatory immune signaling is seen in myelodysplastic syndromes with SF3B1 mutations
Source: eLife. 2022 Aug 30;11:e78136. doi: 10.7554/eLife.78136 (PMC9427103; doi:10.7554/eLife.78136)

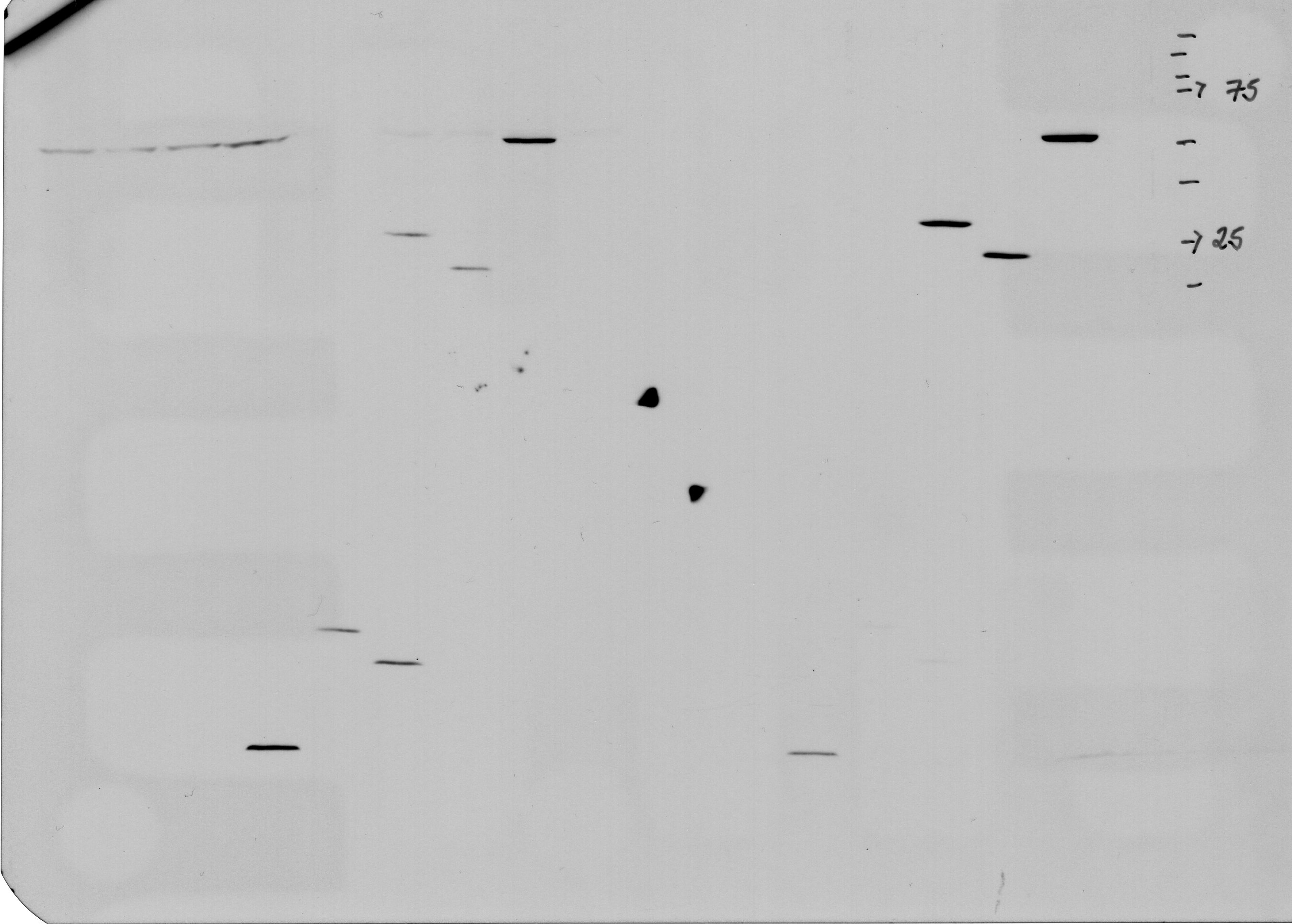

Supplement: Figure 2—source data 1. [file elife-78136-fig2-data1.zip › Figure 2 -Source data 1/1.jpg]

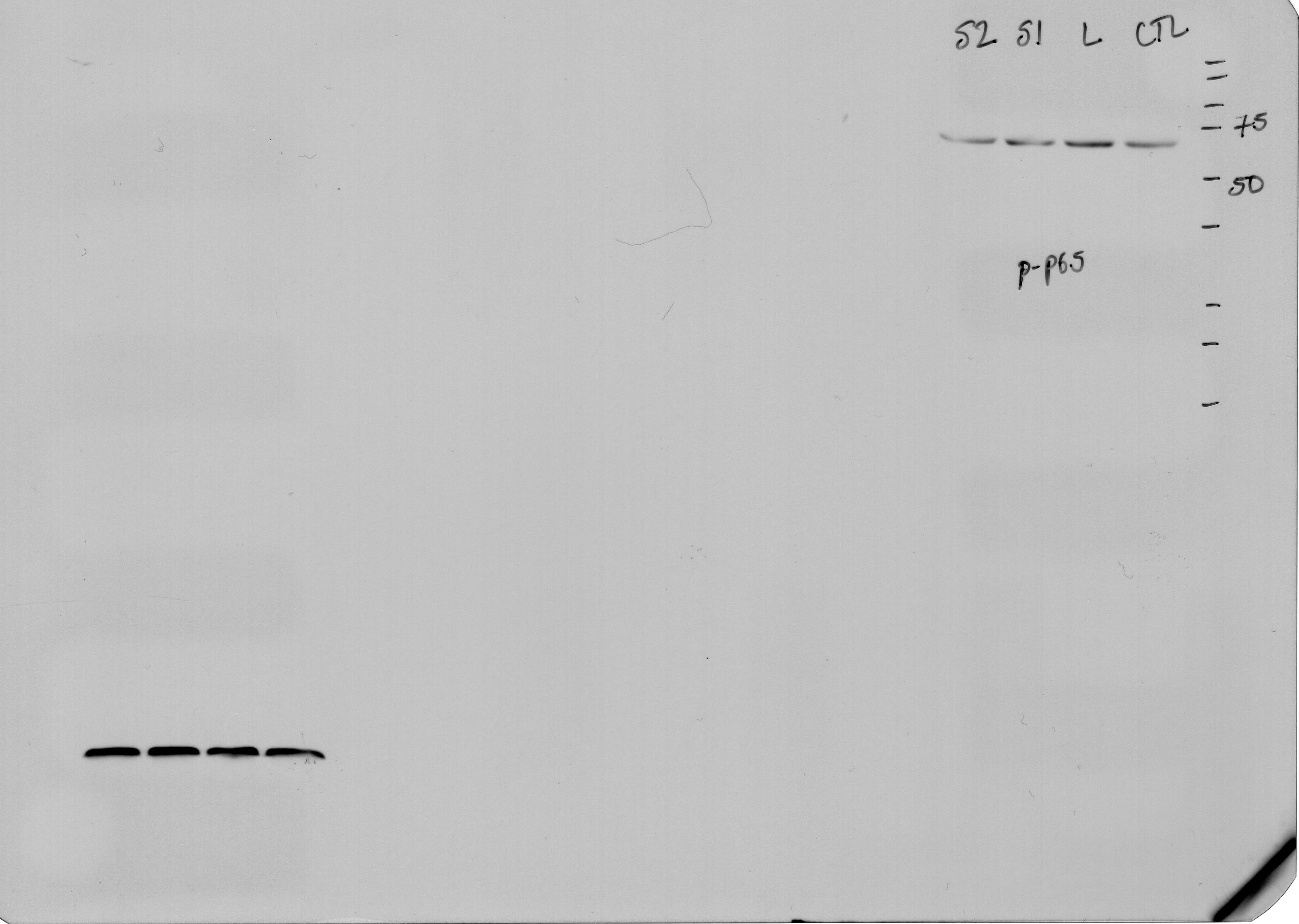

Supplement: Figure 2—source data 1. [file elife-78136-fig2-data1.zip › Figure 2 -Source data 1/2.jpg]

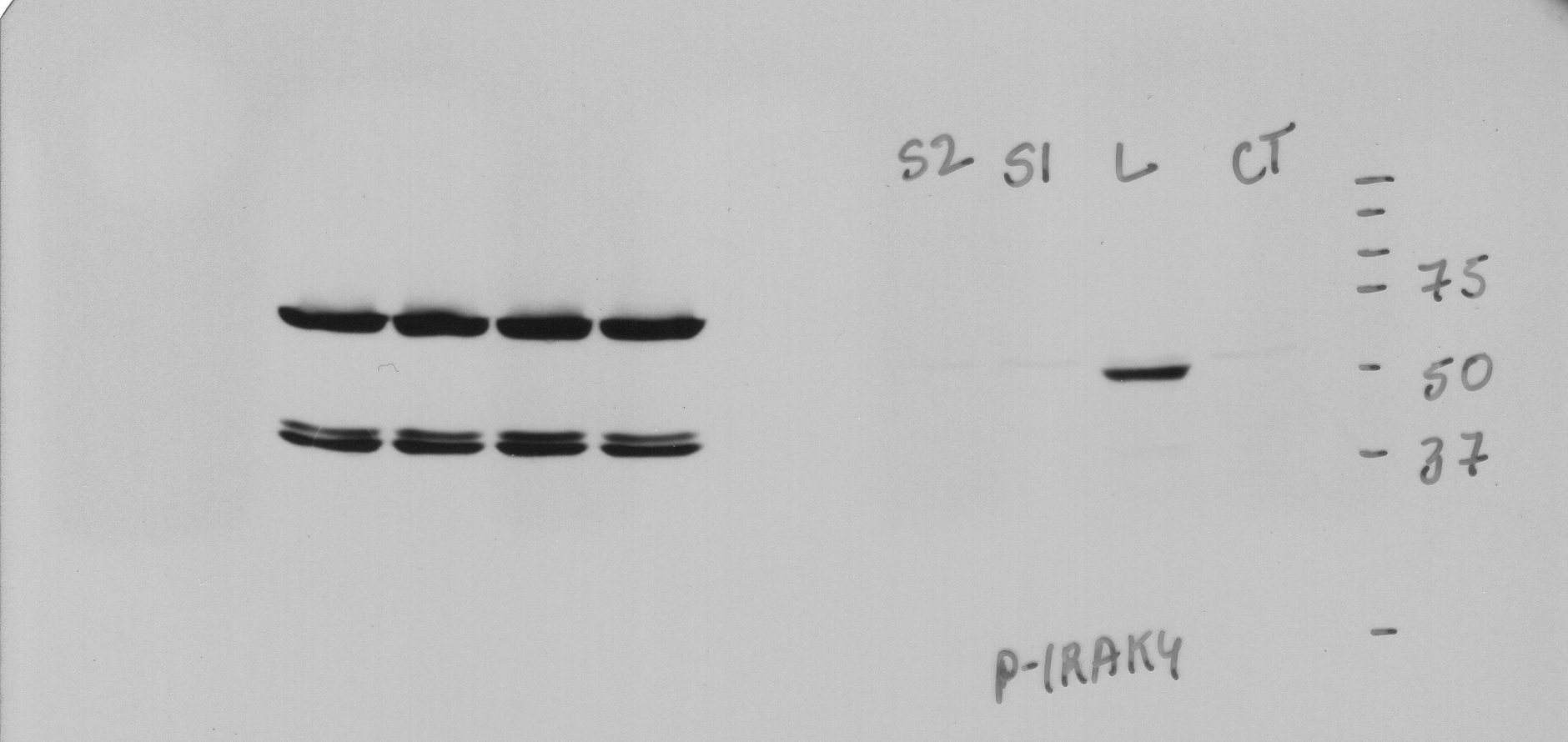

Supplement: Figure 2—source data 1. [file elife-78136-fig2-data1.zip › Figure 2 -Source data 1/3.jpg]

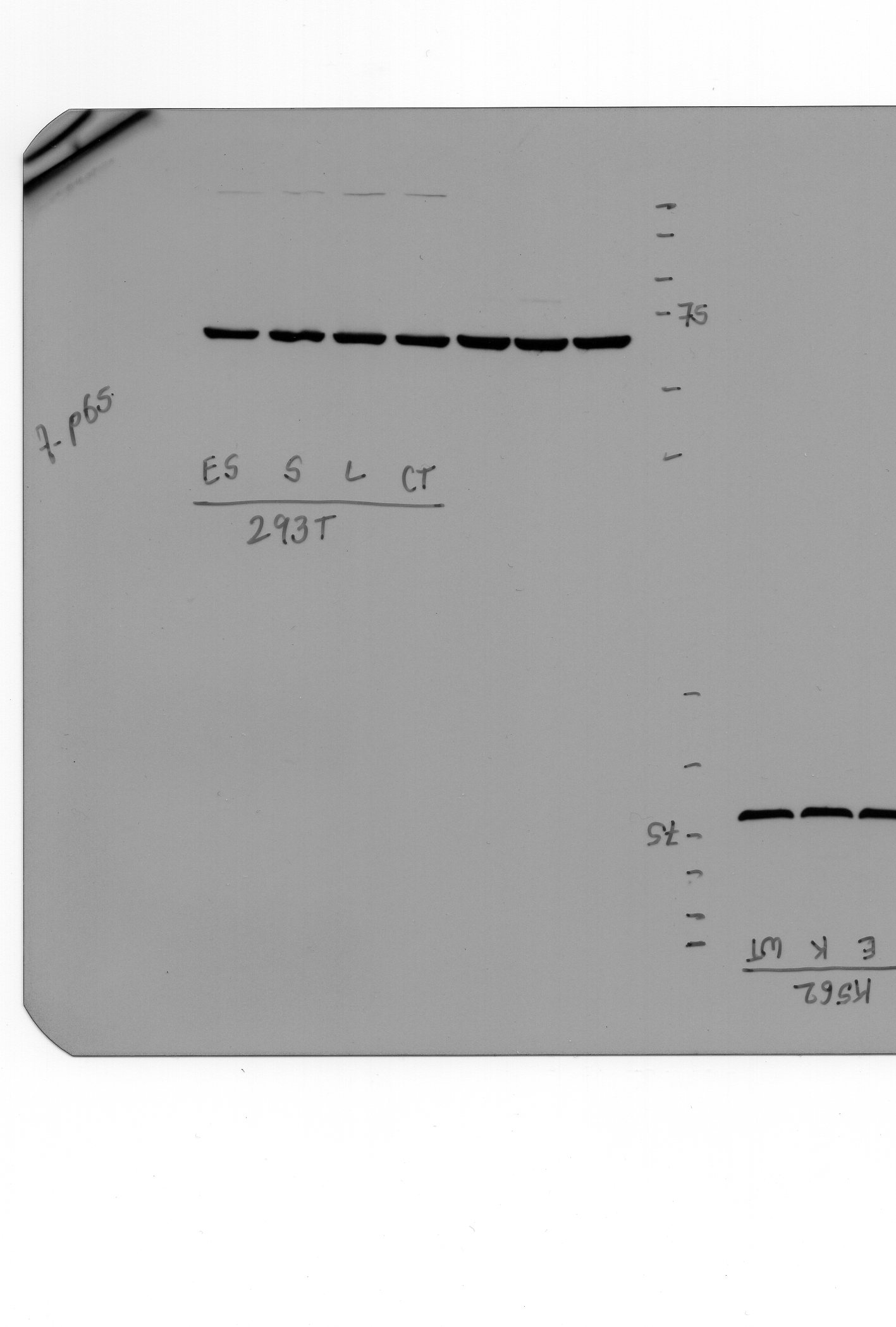

Supplement: Figure 2—source data 1. [file elife-78136-fig2-data1.zip › Figure 2 -Source data 1/4.jpg]

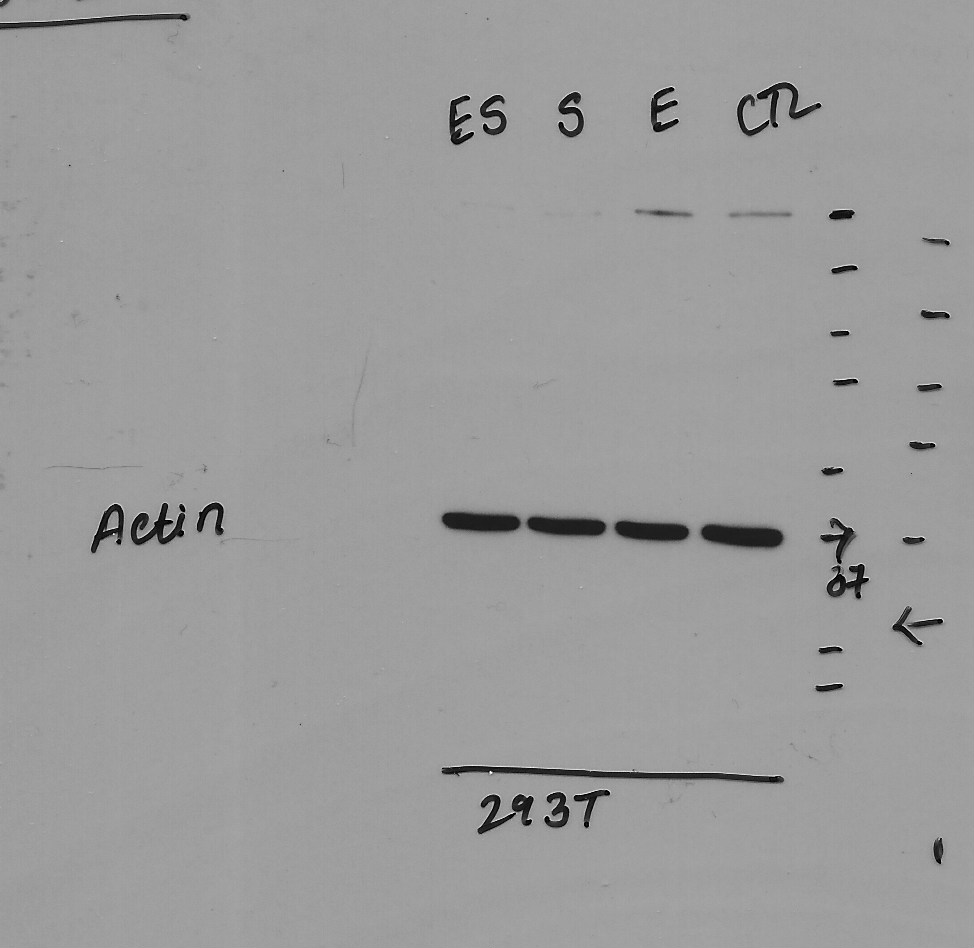

Supplement: Figure 2—source data 1. [file elife-78136-fig2-data1.zip › Figure 2 -Source data 1/Fig 2 B actin.jpeg]

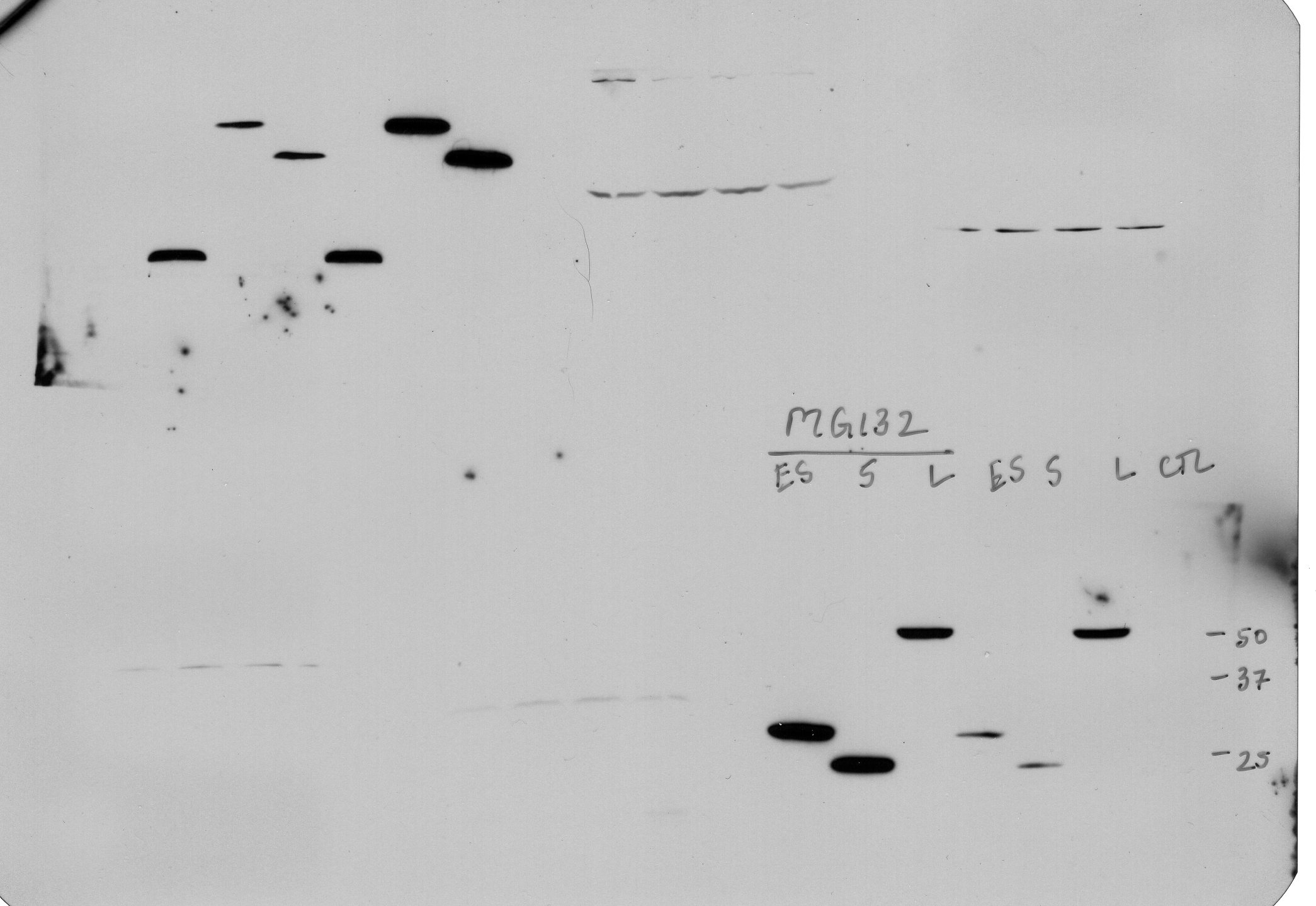

Supplement: Figure 2—source data 2. [file elife-78136-fig2-data2.zip › Figure 2 -Source data 2/img016.jpg]

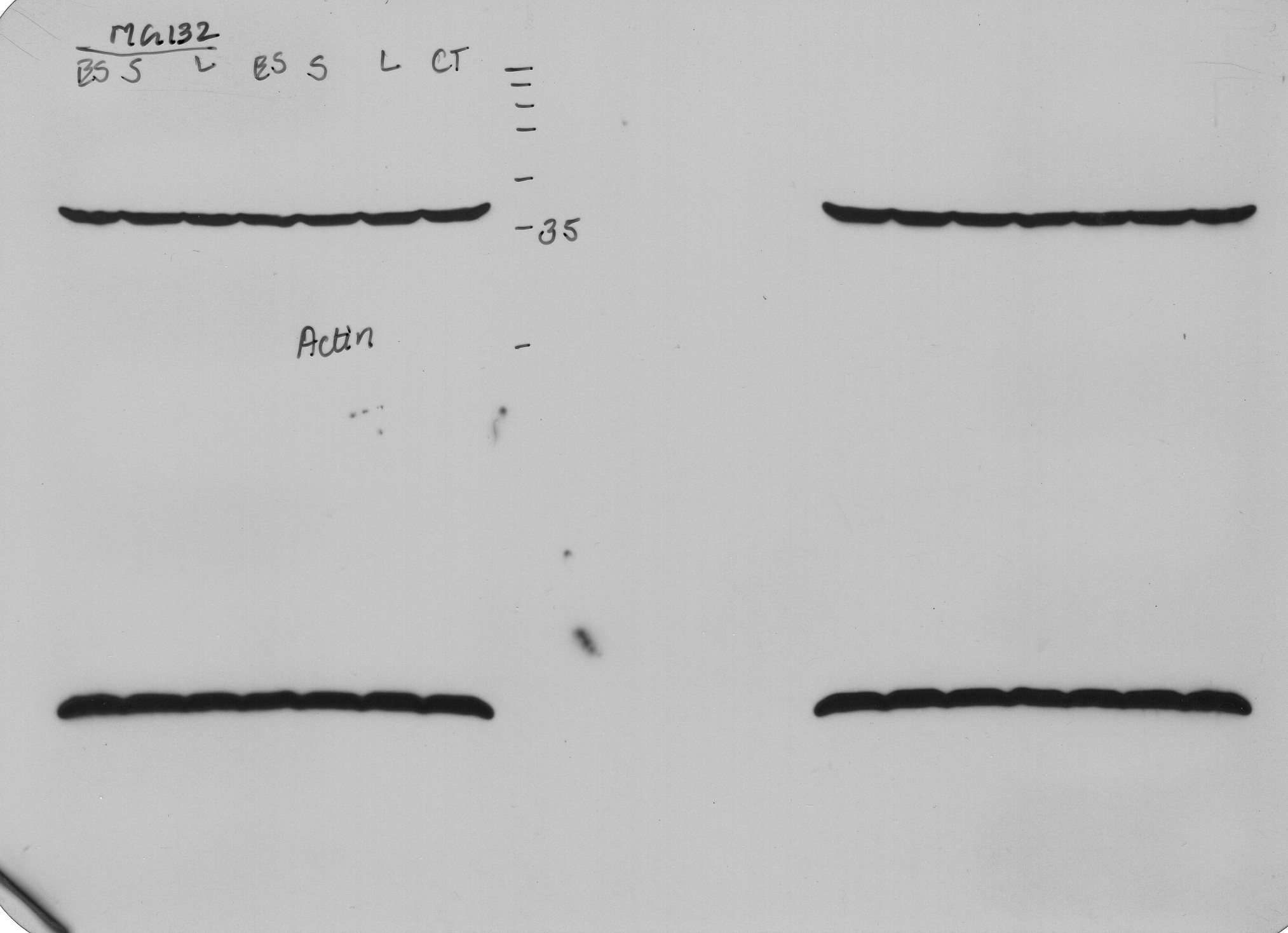

Supplement: Figure 2—source data 2. [file elife-78136-fig2-data2.zip › Figure 2 -Source data 2/img017.jpg]

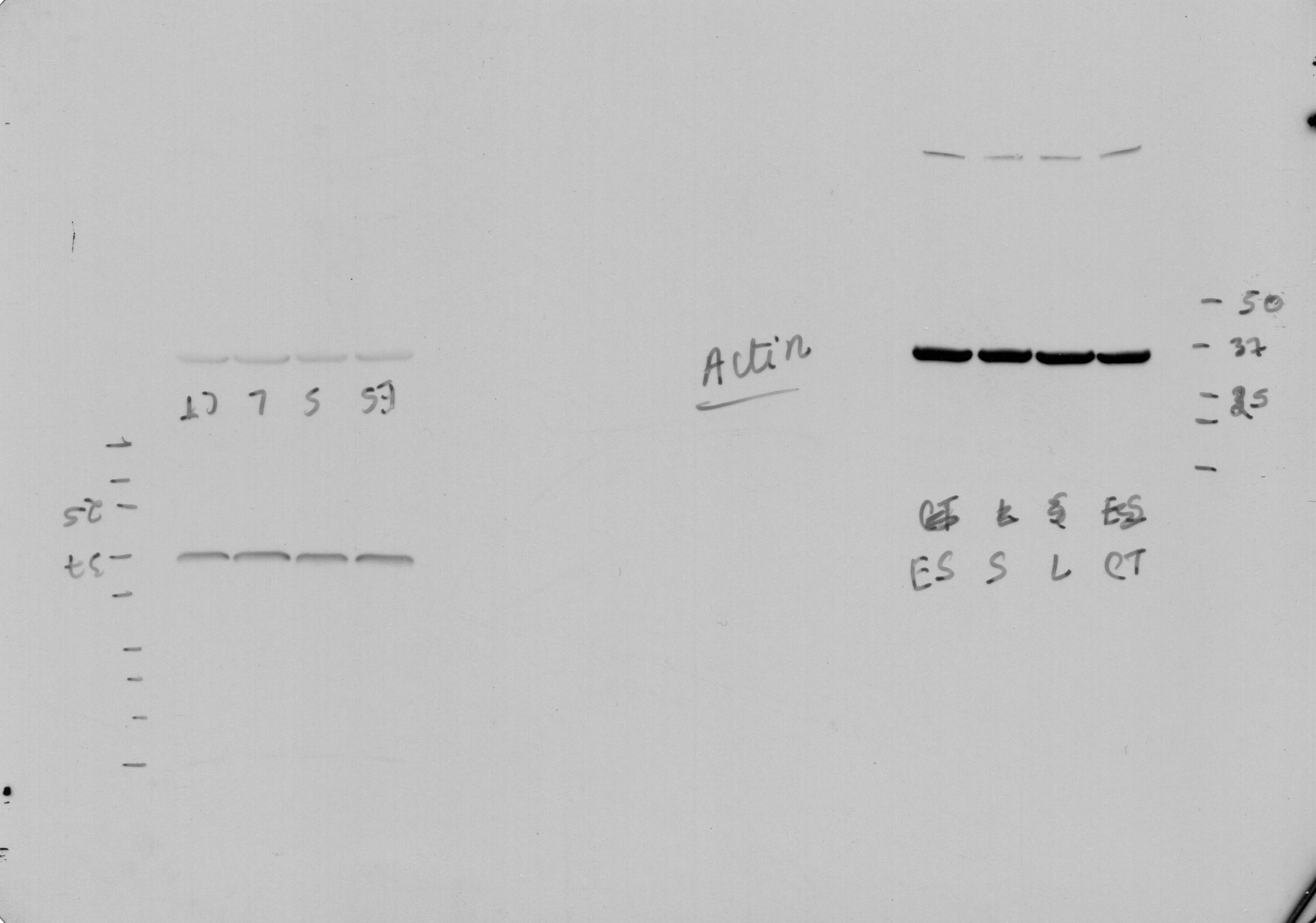

Supplement: Figure 2—source data 3. [file elife-78136-fig2-data3.zip › Figure 2 -Source data 3/img038.jpg]

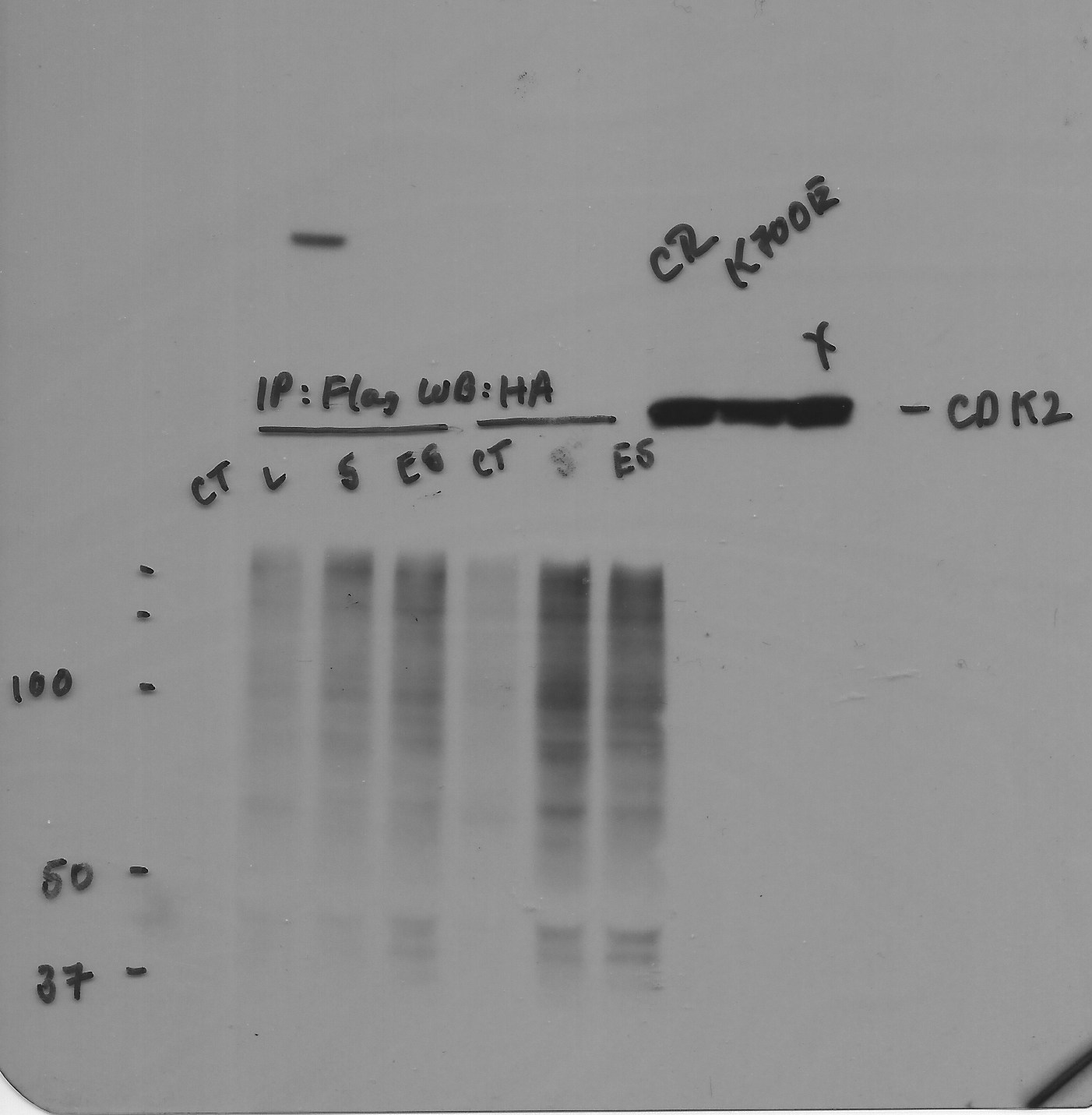

Supplement: Figure 2—source data 3. [file elife-78136-fig2-data3.zip › Figure 2 -Source data 3/L S ES IP Flag WB HA 48 and 63 together 1.jpeg]

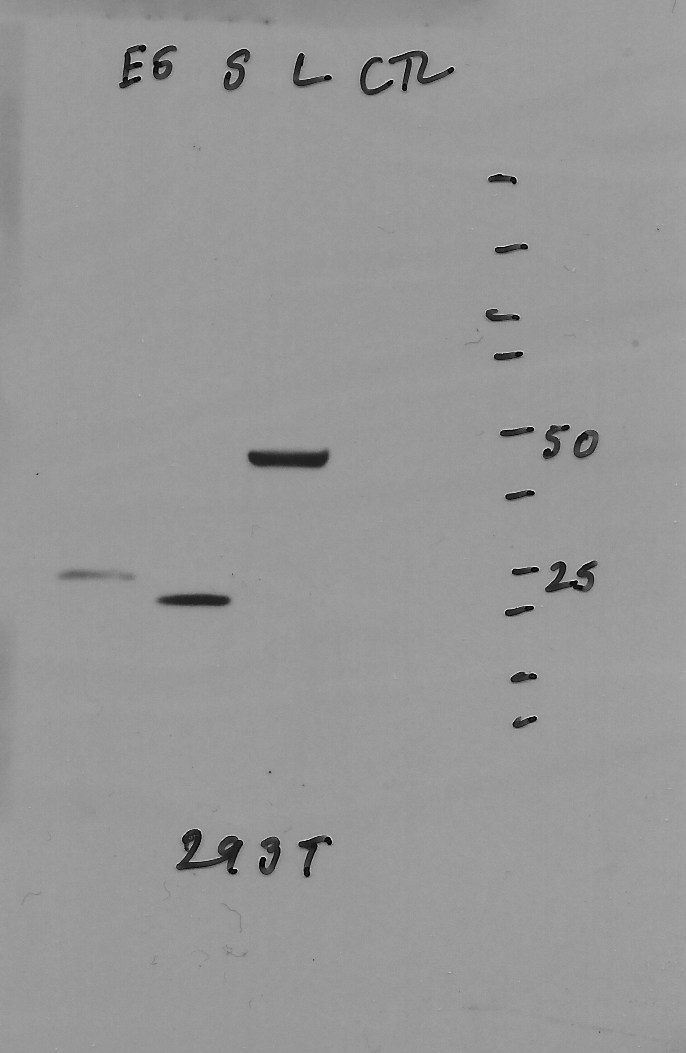

Supplement: Figure 2—source data 3. [file elife-78136-fig2-data3.zip › Figure 2 -Source data 3/L S ES IP Flag WB HA Input.jpeg]

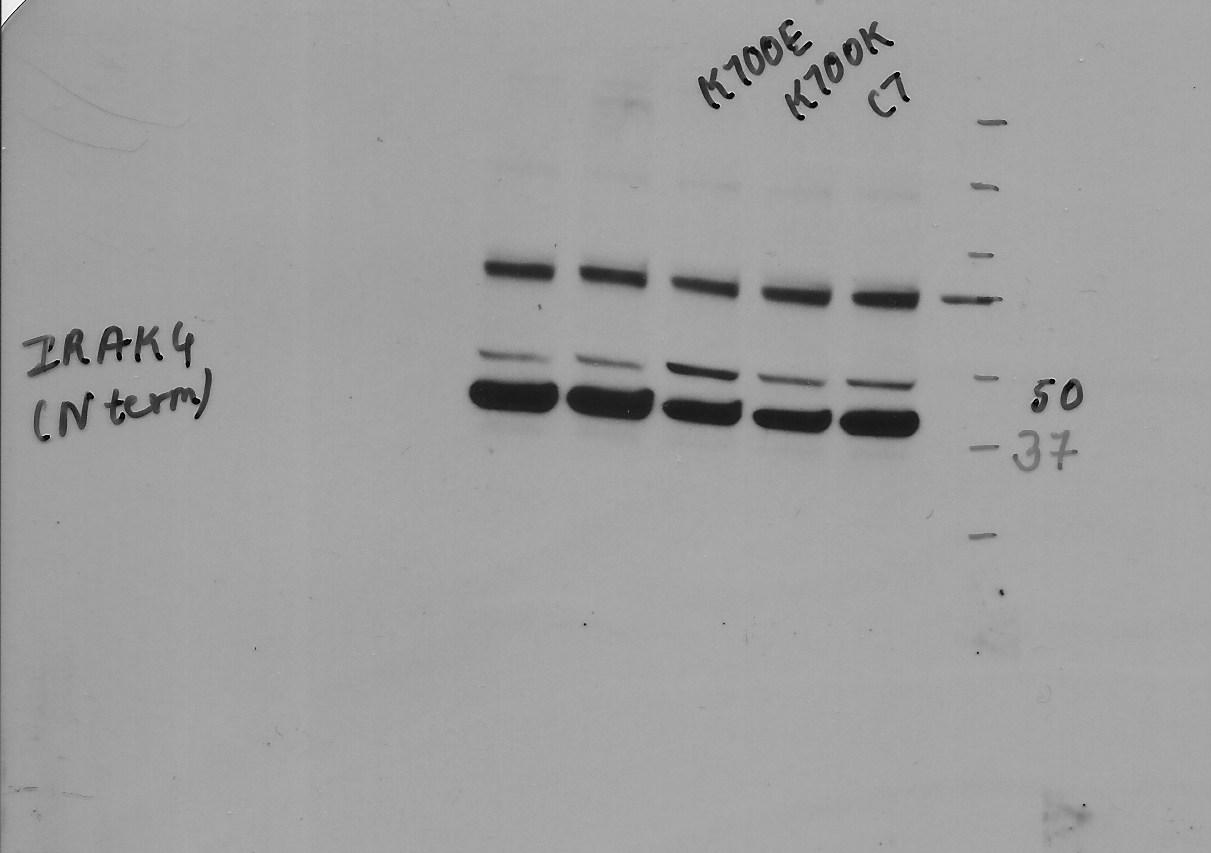

Supplement: Figure 2—source data 4. [file elife-78136-fig2-data4.zip › Figure 2 -Source data 4/1.jpeg]

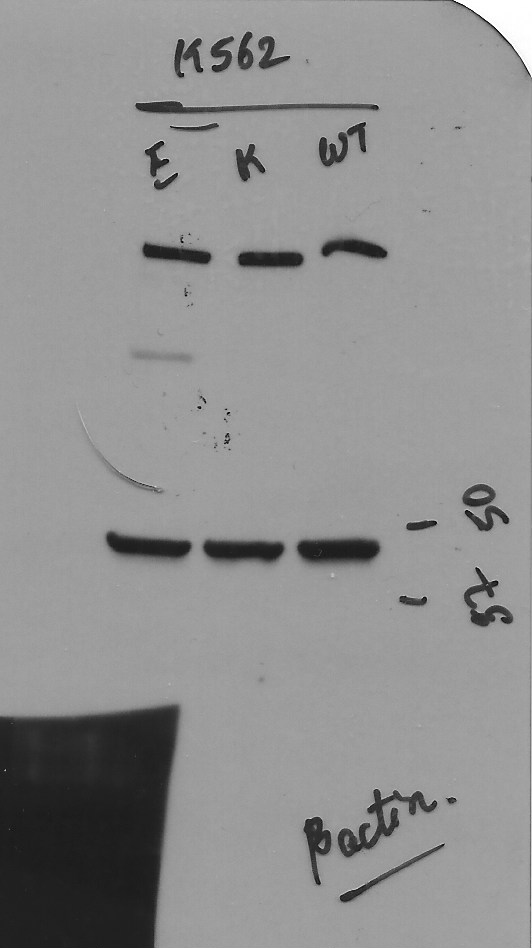

Supplement: Figure 2—source data 4. [file elife-78136-fig2-data4.zip › Figure 2 -Source data 4/Fig 2 E actin.jpeg]

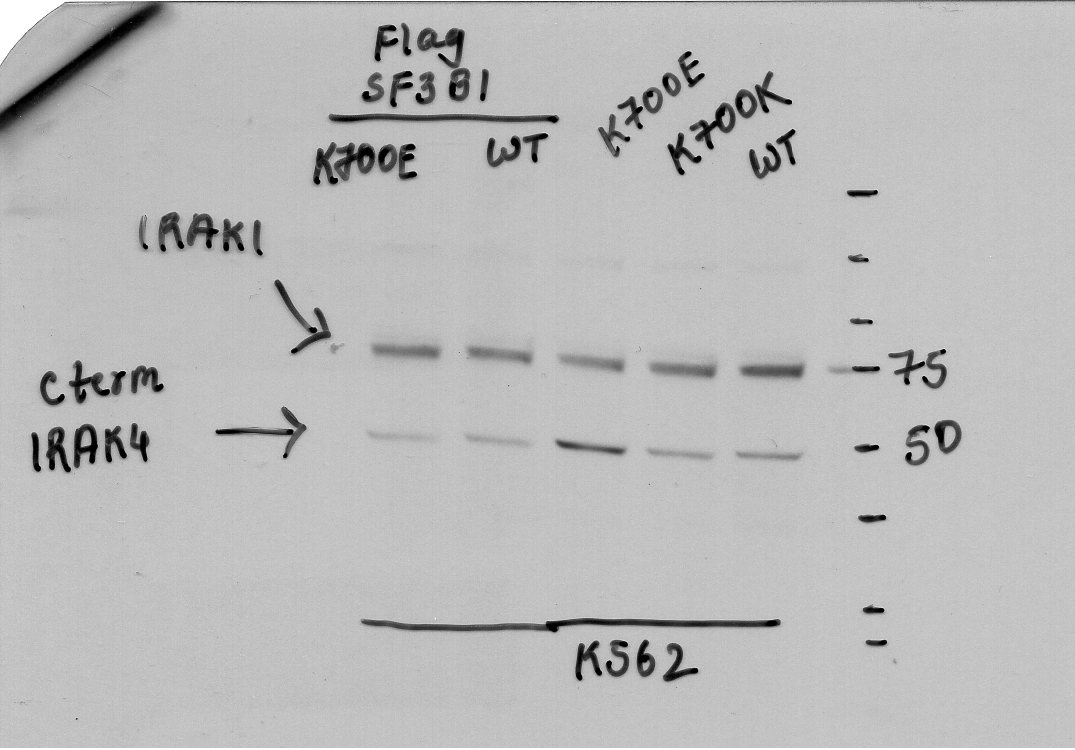

Supplement: Figure 2—source data 4. [file elife-78136-fig2-data4.zip › Figure 2 -Source data 4/img032.jpg]

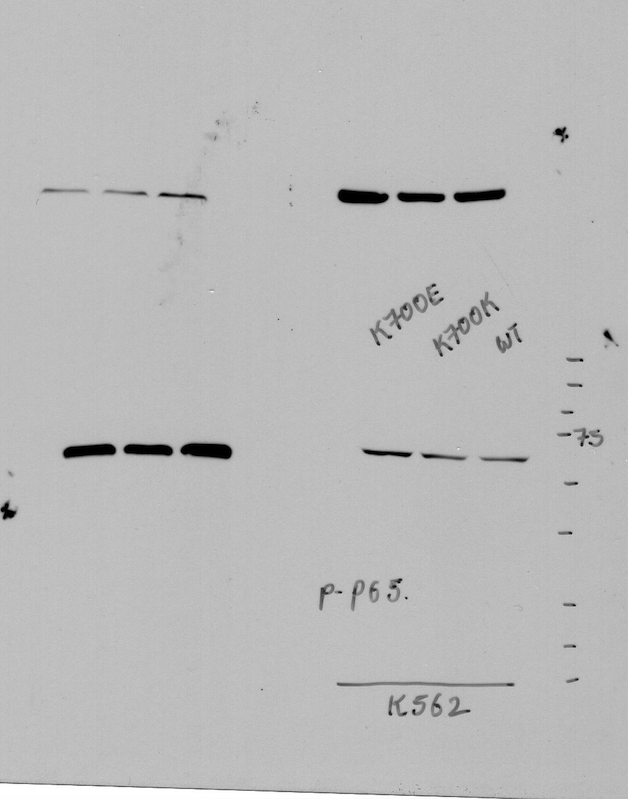

Supplement: Figure 2—source data 4. [file elife-78136-fig2-data4.zip › Figure 2 -Source data 4/p-p65 K562 CT, WT, K700E and total p65.png]

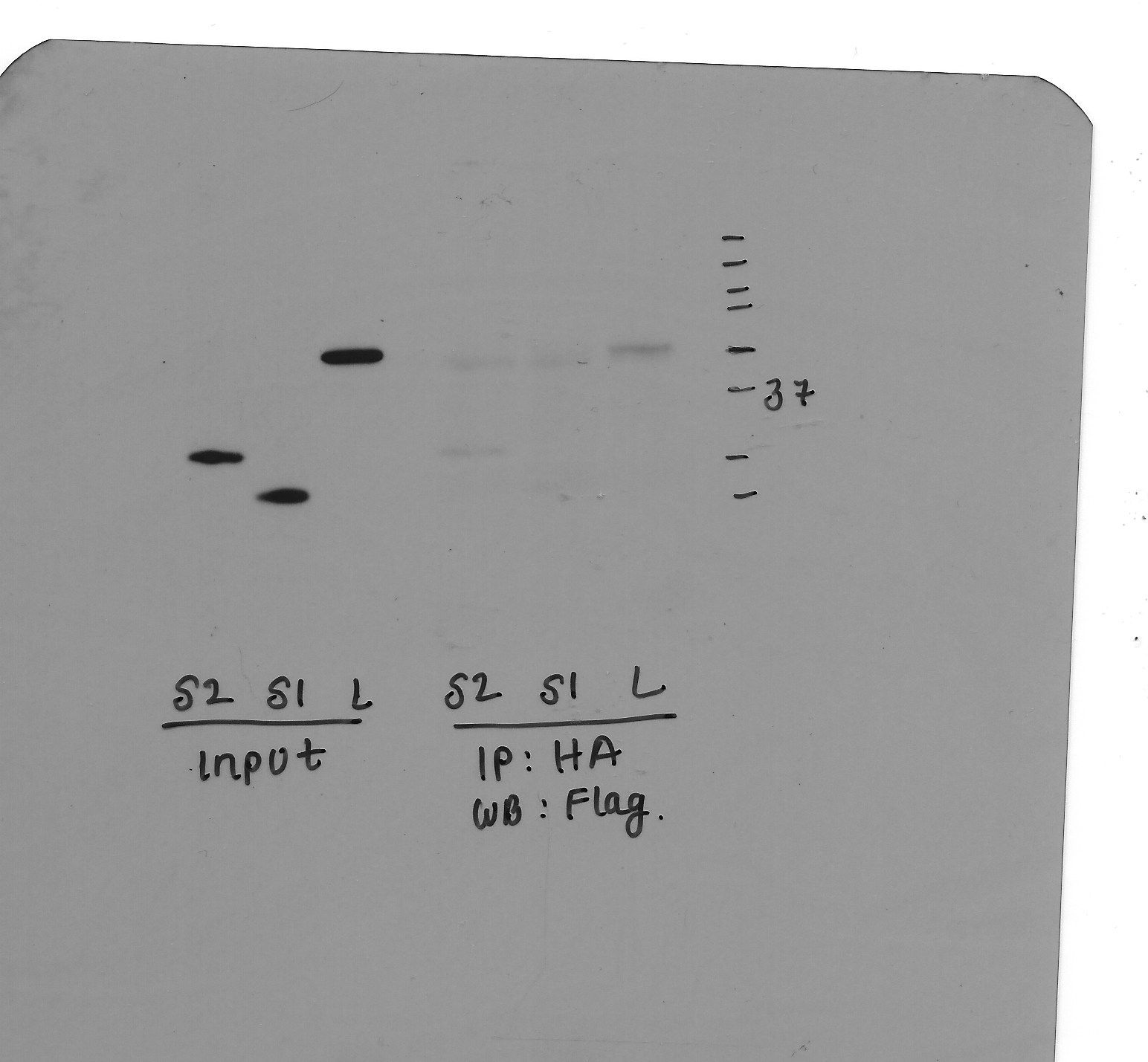

Supplement: Figure 2—source data 5. [file elife-78136-fig2-data5.zip › Figure 2 -Source data 5/01-19-21 2.jpeg]

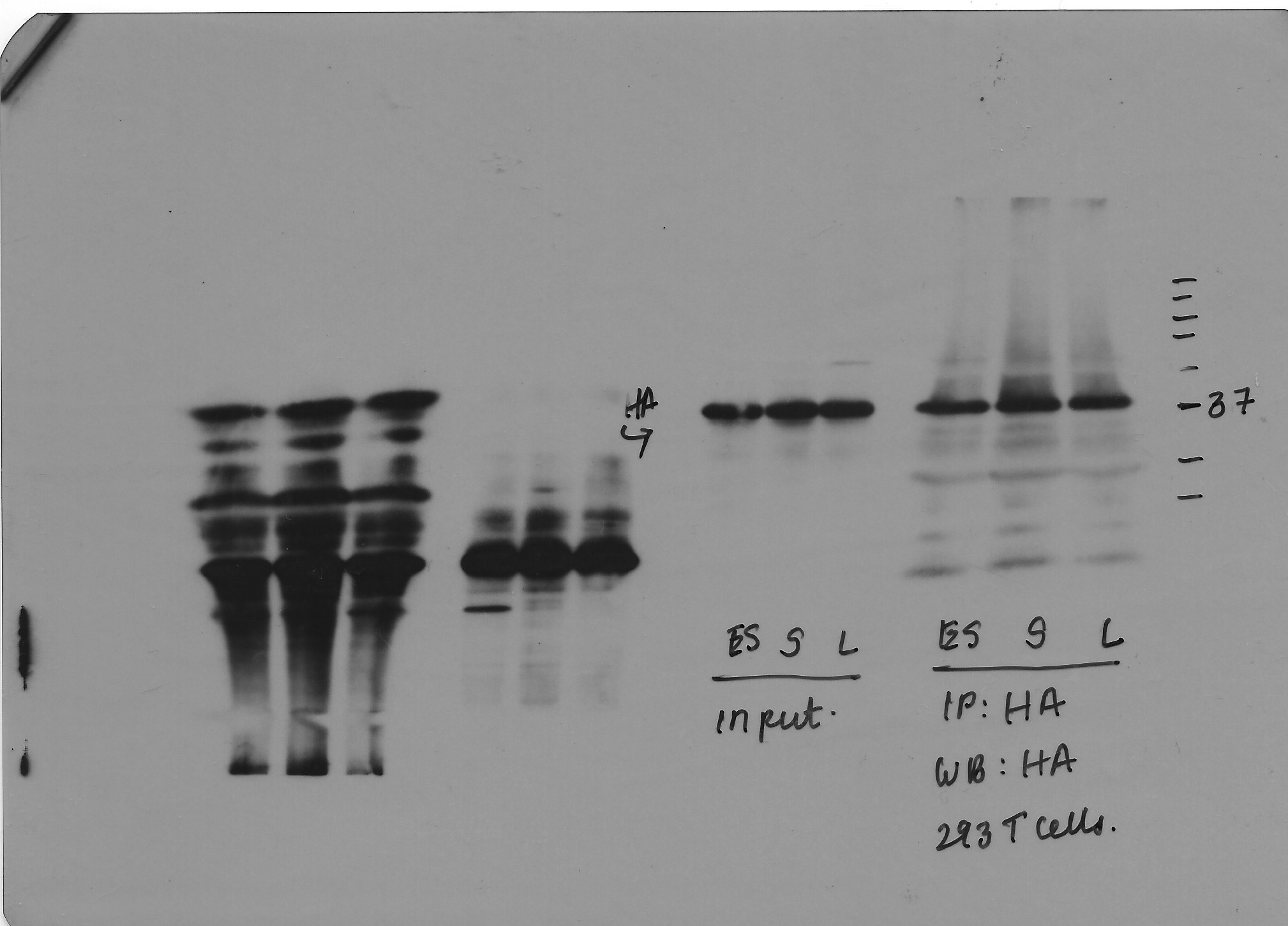

Supplement: Figure 2—source data 5. [file elife-78136-fig2-data5.zip › Figure 2 -Source data 5/01-19-21 4.jpeg]

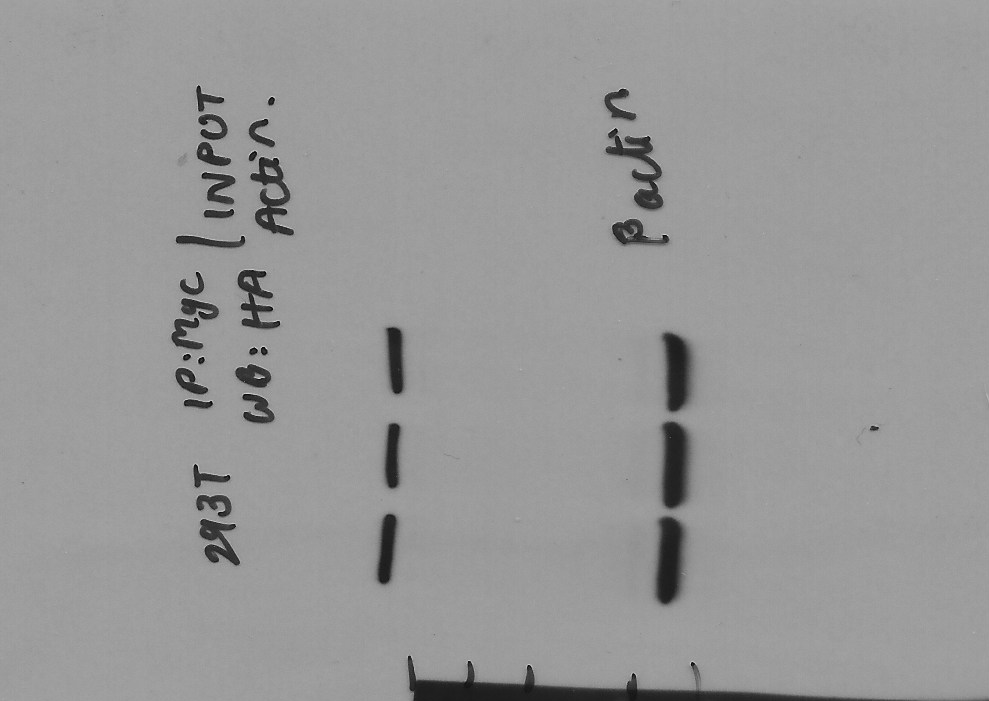

Supplement: Figure 3—source data 1. [file elife-78136-fig3-data1.zip › Figure 3 -Source data 1/Fig 3 C actin.jpeg]

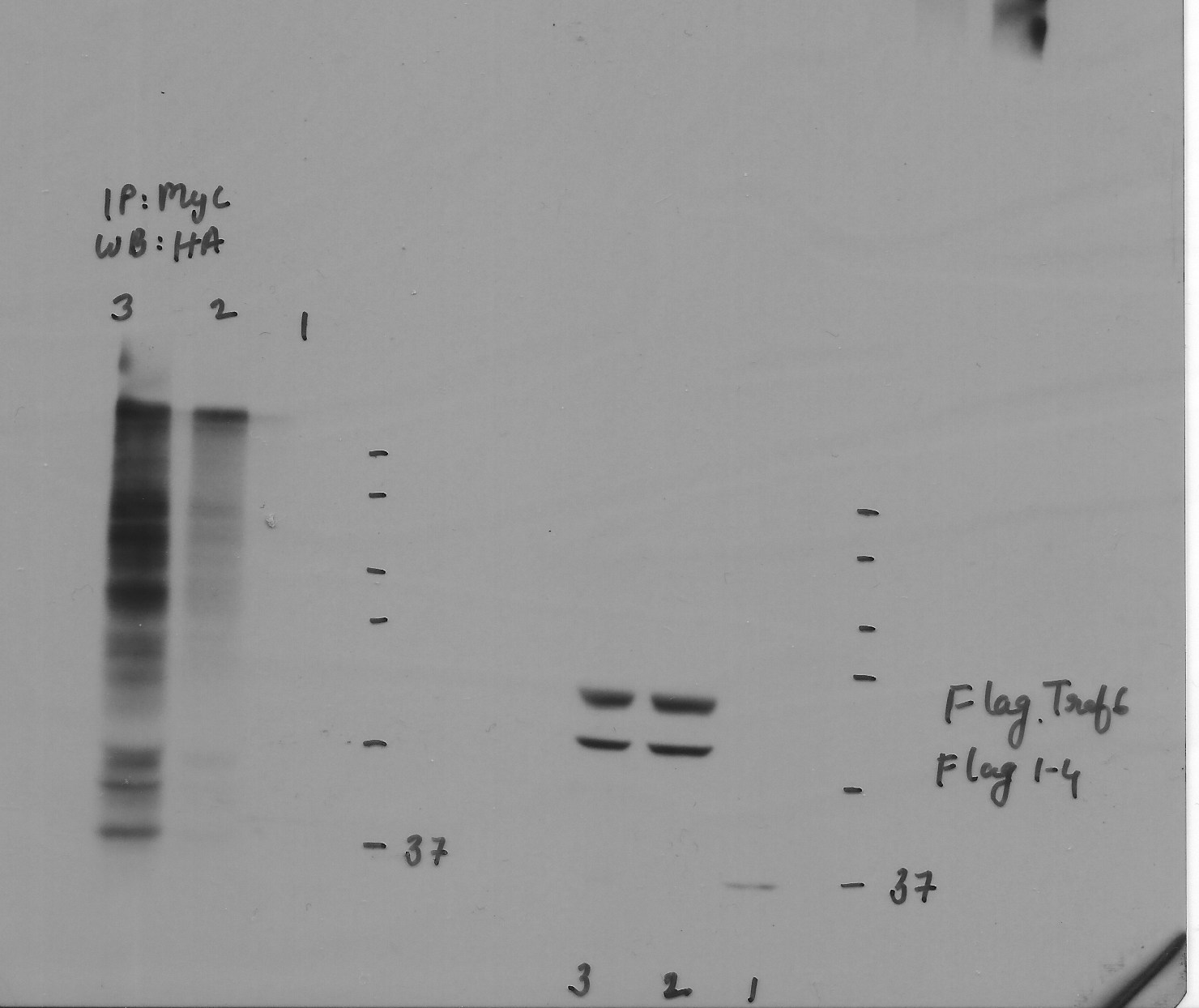

Supplement: Figure 3—source data 1. [file elife-78136-fig3-data1.zip › Figure 3 -Source data 1/Fig 3C IP Myc IB HA and Inputs.jpeg]

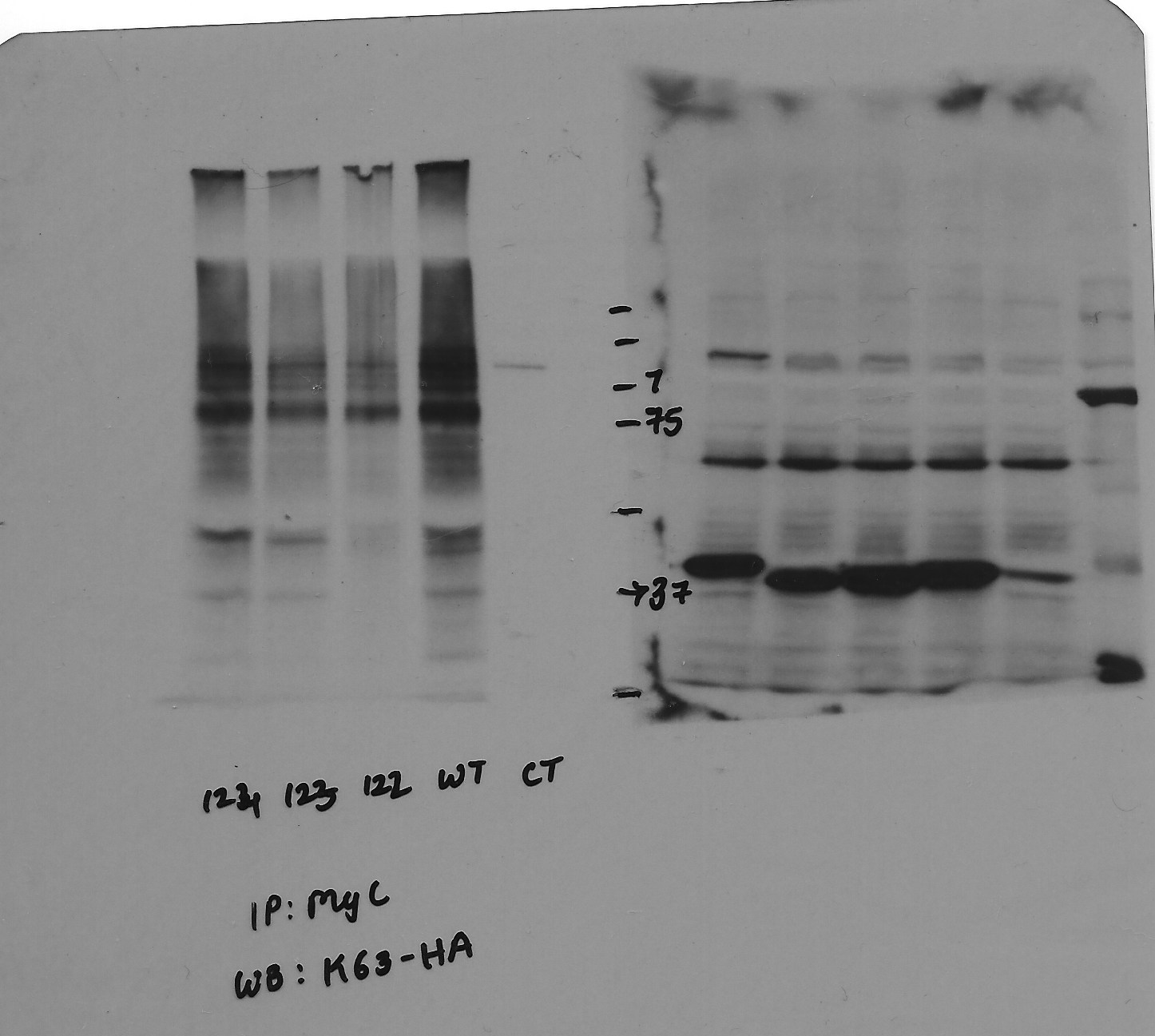

Supplement: Figure 3—source data 2. [file elife-78136-fig3-data2.zip › Figure 3 -Source data 2/01-19-21.jpeg]

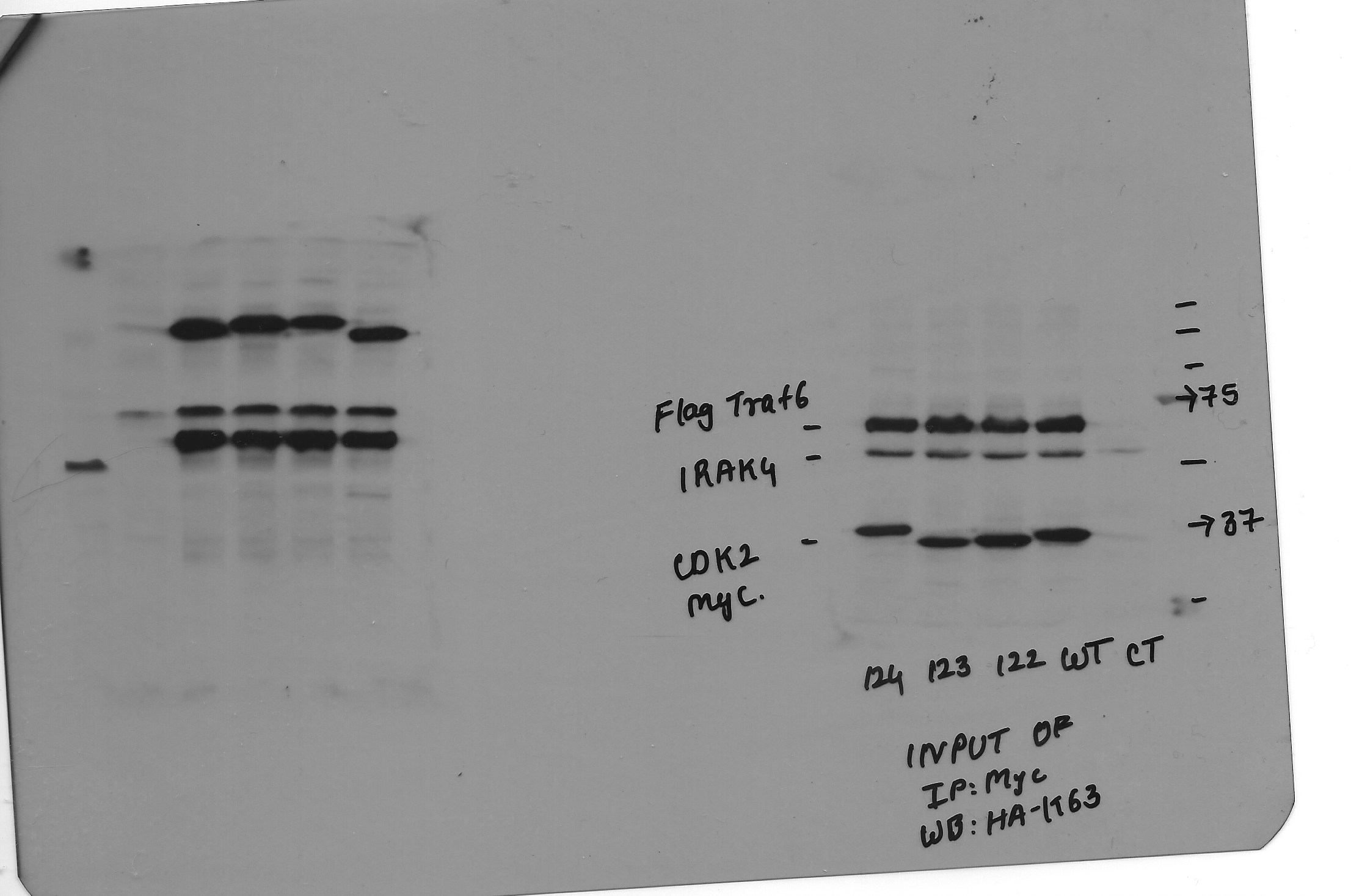

Supplement: Figure 3—source data 2. [file elife-78136-fig3-data2.zip › Figure 3 -Source data 2/Traf6 I-4 CDK2 input.jpeg]

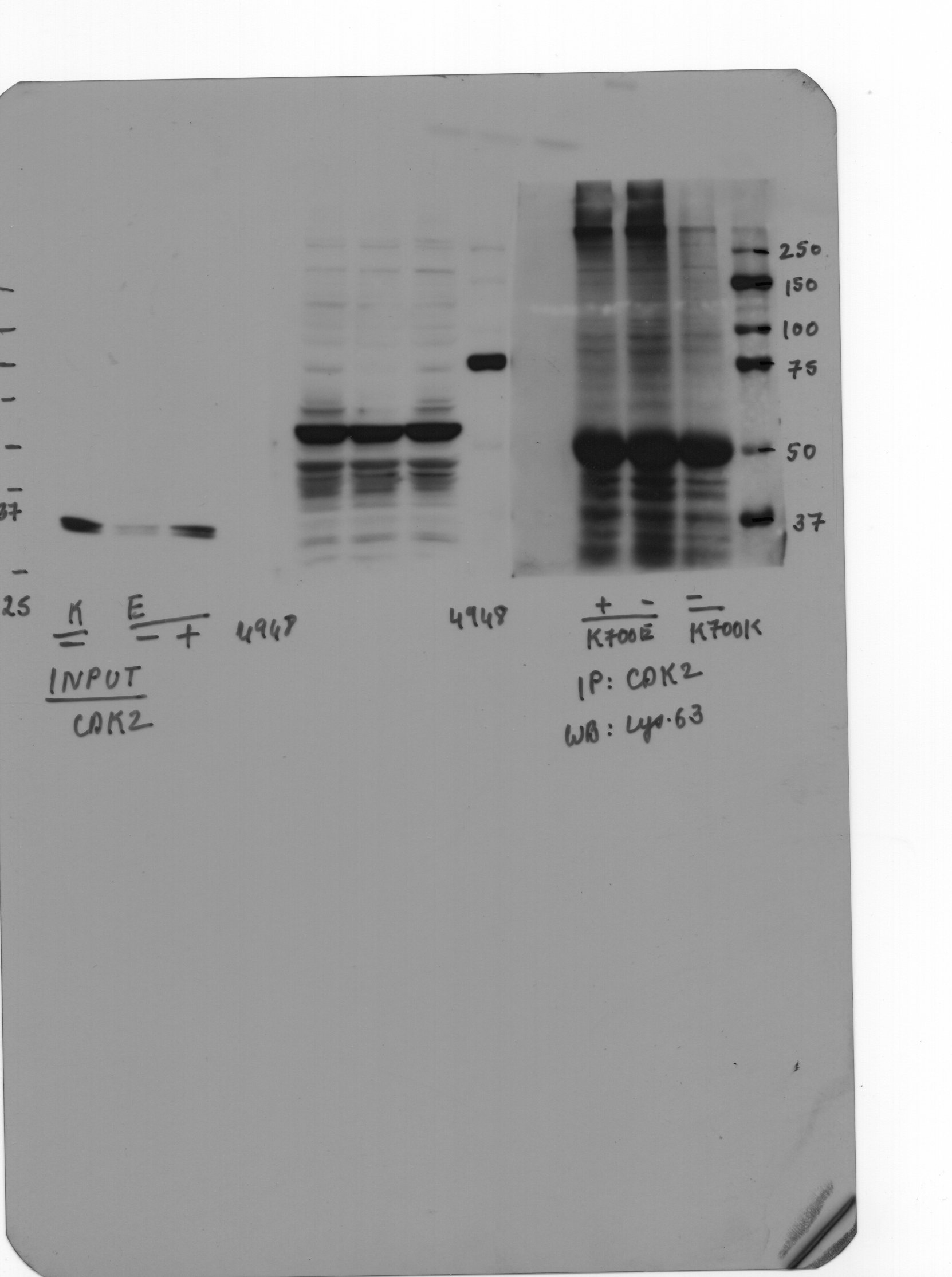

Supplement: Figure 3—source data 3. [file elife-78136-fig3-data3.zip › Figure 3 -Source data 3/IP CDK2 WB 63 and Input.jpg]

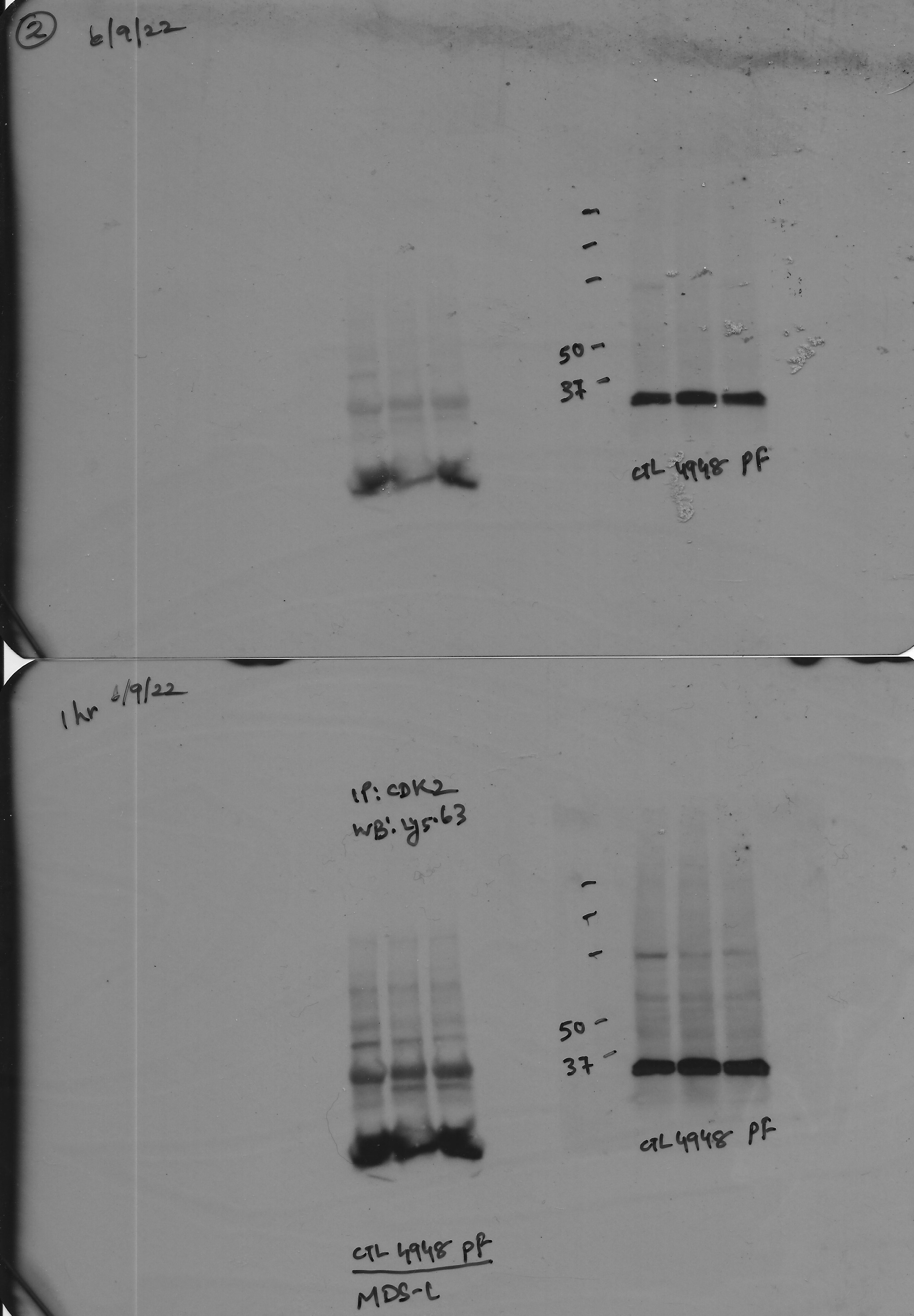

Supplement: Figure 3—figure supplement 1—source data 1. [file elife-78136-fig3-figsupp1-data1.zip › Figure 3-figure supplement 1-Source data 1/Figure 3-figure supplement 1-Source data 1.jpeg]
