# Supplementary material for: Activation of targetable inflammatory immune signaling is seen in myelodysplastic syndromes with SF3B1 mutations
Source: eLife. 2022 Aug 30;11:e78136. doi: 10.7554/eLife.78136 (PMC9427103; doi:10.7554/eLife.78136)
Supplement: Supplementary file 1. [file elife-78136-supp1.pptx]

## Slide 1
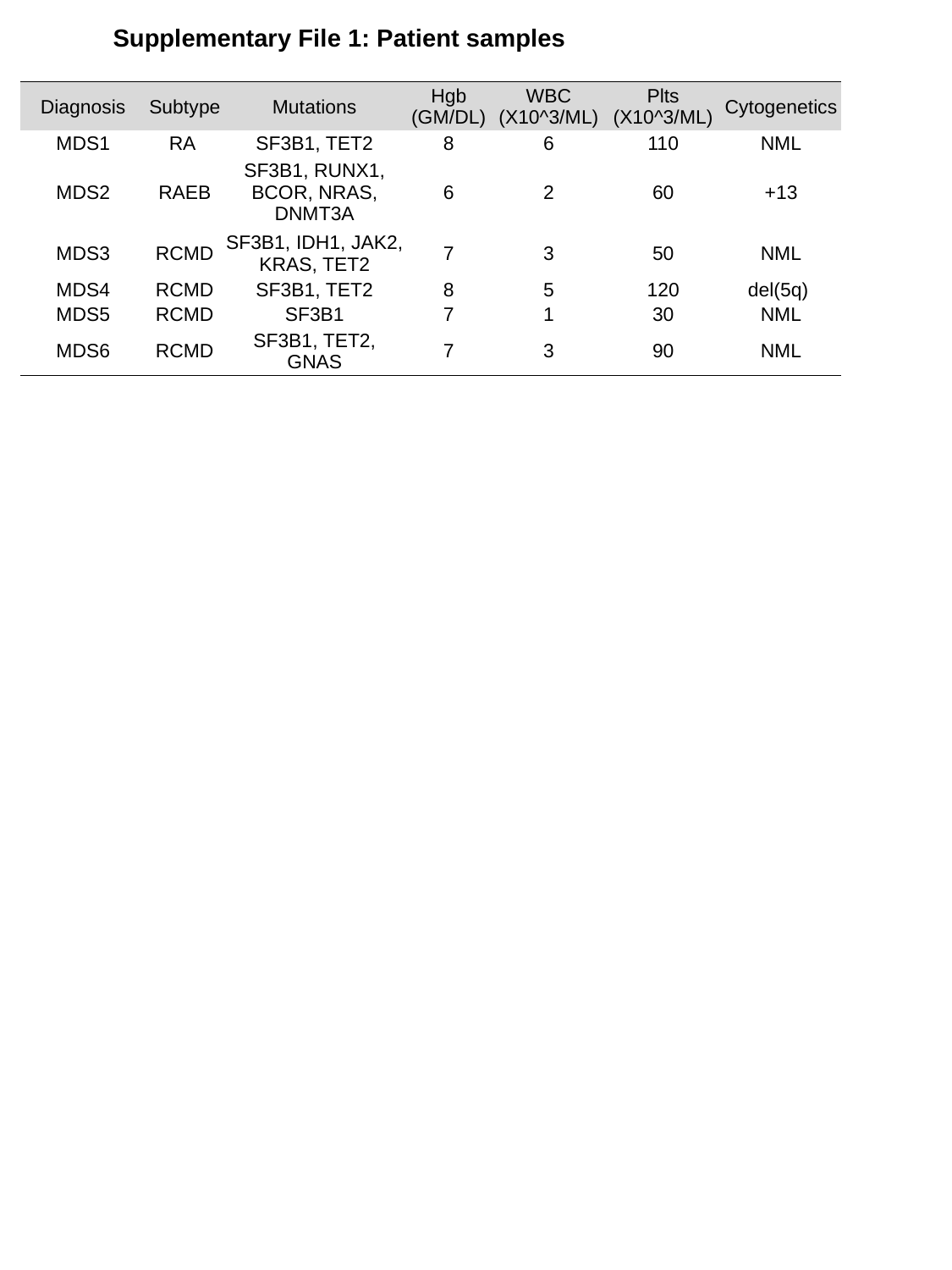

# Supplementary File 1: Patient samples
| Diagnosis | Subtype | Mutations | Hgb (GM/DL) | WBC (X10^3/ML) | Plts (X10^3/ML) | Cytogenetics |
| --- | --- | --- | --- | --- | --- | --- |
| MDS1 | RA | SF3B1, TET2 | 8 | 6 | 110 | NML |
| MDS2 | RAEB | SF3B1, RUNX1, BCOR, NRAS, DNMT3A | 6 | 2 | 60 | +13 |
| MDS3 | RCMD | SF3B1, IDH1, JAK2, KRAS, TET2 | 7 | 3 | 50 | NML |
| MDS4 | RCMD | SF3B1, TET2 | 8 | 5 | 120 | del(5q) |
| MDS5 | RCMD | SF3B1 | 7 | 1 | 30 | NML |
| MDS6 | RCMD | SF3B1, TET2, GNAS | 7 | 3 | 90 | NML |
